# Evaluation of activities of daily living using an electronic version of the Longshi Scale in patients with stroke: reliability, consistency, and preference

**DOI:** 10.1186/s12911-024-02508-0

**Published:** 2024-05-15

**Authors:** Kaiwen Xue, Weihao Li, Fang Liu, Xiangxiang Liu, John Wong, Mingchao Zhou, Chunli Cai, Jianjun Long, Jiehui Li, Zeyu Zhang, Weilin Hou, Guohui Nie, Yulong Wang

**Affiliations:** 1 Department of Rehabilitation, The Second People’s Hospital of Shenzhen, The First Affiliated Hospital of Shenzhen University Health Science Centre, Shenzhen, China; 2 National Clinical Research Center for Infectious Disease of Shenzhen; Shenzhen Third People’s Hospital, Shenzhen, China; 3 School of Nursing and Department of Occupational Therapy, MGH Institute of Health Professions, Boston, MA USA; 4 Operation Department, Shenzhen Yilanda Technology Co. Ltd., Shenzhen, China; 5 School of Rehabilitation Medicine, The Shandong University of Traditional Chinese Medicine, Shandong, China; 6 Department of Rehabilitation, Changzhou Hospital of Traditional Chinese Medicine, Jiangsu, China

**Keywords:** Longshi Scale, Activities of daily living, Electronic version, WeChat version, Stroke

## Abstract

**Background:**

The Longshi Scale is a pictorial assessment tool for evaluating activities of daily living (ADL) in patients with stroke. The paper-based version presents challenges; thus, the WeChat version was created to enhance accessibility. Herein, we aimed to validate the inter-rater and test–retest reliabilities of the WeChat version of the Longshi Scale and explore its potential clinical applications.

**Methods:**

We recruited 115 patients with stroke in the study. The ADL results of each patient were assessed using both the WeChat and paper-based version of the Longshi Scale; each evaluation was conducted by 28 health professionals and 115 caregivers separately. To explore the test–retest reliability of the WeChat version, 22 patients were randomly selected and re-evaluated by health professionals using the WeChat version. All evaluation criteria were recorded, and all evaluators were surveyed to indicate their preference between the two versions.

**Results:**

Consistency between WeChat and the paper-based Longshi Scale was high for ADL scores by health professionals (ICC_2,1_ = 0.803–0.988) and caregivers (ICC_2,1_ = 0.845–0.983), as well as for degrees of disability (κw = 0.870 by professionals; κw = 0.800 by caregivers). Bland–Altman analysis showed no significant discrepancies. The WeChat version exhibited good test–retest reliability (κw = 0.880). The WeChat version showed similar inter-rater reliability in terms of the ADL score evaluated using the paper-based version (ICC_2,1_ = 0.781–0.941). The time to complete assessments did not differ significantly, although the WeChat version had a shorter information entry time (*P* < 0.001, 95% confidence interval: –43.463 to –15.488). Health professionals favored the WeChat version (53.6%), whereas caregivers had no significant preference.

**Conclusions:**

The WeChat version of the Longshi Scale is reliable and serves as a suitable alternative for health professionals and caregivers to assess ADL levels in patients with stroke. The WeChat version of the Longshi Scale is considered user-friendly by health professionals, although it is not preferred by caregivers.

**Trial registration:**

This study was approved by the Ethics Committee of the Second People’s Hospital of Shenzhen (approval number: 20210812003-FS01) and registered on the Clinical Trial Register Center website: clinicaltrials.gov on January 31, 2022 (registration no.: NCT05214638).

## Background

The Longshi Scale is an innovative tool to assess activities of daily living (ADL) in individuals with disabilities, especially in patients with stroke in the Chinese context. The Longshi Scale is a pictorial tool that facilitates a more standardized, regulated, and easily understandable assessment process. Developed in 2013 based on a survey of individuals with physical disabilities, it categorizes participants into bedridden, domestic, and community groups by querying whether they can independently get out of bed or go outdoors [1]. Previous studies have demonstrated the reliability and validity of the Longshi Scale, with an intraclass correlation coefficient (based on two-way random effects, i.e., ICC_2,1_) of > 0.850 [1], and have shown a strong positive correlation with the Barthel Index (*r* = 0.868) [2]. Although the Longshi Scale is not considered the gold standard for ADL assessment worldwide, it is now recommended as one of the national standards in China (License Code: GB/T 37103–2018) for evaluating functional independence and disabilities [3]. Currently, both the English and Chinese versions of the Longshi Scale are available in electronic and paper-based forms.

The paper-based version of the Longshi Scale, though widely used in clinical settings, poses the following challenges: i) the use of a hard copy during evaluation hinders disinfection, and ii) the process of transferring the obtained data to a computer is time-consuming and prone to errors [4, 5]. In areas with insufficient rehabilitation resources, inadequate facilities may hinder the availability of printing paper [6].

Digital health has gained popularity in recent years owing to its numerous advantages. In the late 1990s, health officials in the United States predicted a transition toward electronic health management and made investments under the Health Information Technology for Economic and Clinical Health Act of 2009 [7]. In Europe, electronic health management has yielded significant results in managing acute and chronic diseases [8]. In the Americas, even in low-to-middle-income countries, the importance of innovations in health systems has been emphasized. Consequently, the use of eHealth systems now exceeds 60% [9]. Beyond Europe and the Americas, the quantity and scope of eHealth systems in low-to-middle-income countries worldwide have also experienced rapid expansion [10].

Digital health can enhance healthcare in rural areas and reduce healthcare disparities between urban and rural settings by offering easy accessibility and providing healthcare providers with clear guidelines [11, 12]. Mobile health includes various healthcare practices and can enhance health promotion and disease prevention by the efficient registration, storage, and processing of large volumes of health data. A recent study identified mobile applications, social media, and wearable devices as the top three technologies that can be integrated into health management [13]. The Rehab Express mobile application was introduced as the electronic version of the Longshi Scale in 2019. However, this initiative was unsuccessful because users expressed dissatisfaction with the requirement to download the application. Moreover, patients and their families/caregivers raised concerns regarding accessibility, as they had to obtain authorization before each use. In early 2021, a new electronic version of the Longshi Scale was developed as an in-application program within WeChat. As of the third quarter of 2023, WeChat had amassed over 1.3 billion users. Although the majority are Chinese, the platform is gaining an increasingly diverse international user base and is currently the world’s fifth most popular social platform [14]. This version offers improved accessibility and convenience, granting all users equal access to the scale for evaluation and review [15].

Given that the mode of question delivery may influence the response, it is necessary to verify both the utility and psychometrics of the WeChat version in comparison with the original paper-based version, as well as the equivalence between the two [16]. Hence, the objectives of the current study were as follows: (1) to evaluate the inter-rater and test–retest reliabilities of the ADL score and the degree of disability, utilizing the WeChat version of the Longshi Scale; and (2) to conduct a comparative analysis of the evaluation duration, time needed to complete basic information, and evaluator preferences between the two versions. Accordingly, we aimed to determine the clinical applications and potential advancements associated with the WeChat version.

## Methods

This descriptive study was conducted in October 2021 at the Rehabilitation Department of the Second People’s Hospital of Shenzhen and its affiliated rehabilitation hospital, the Nan’ao People’s Hospital in Shenzhen, China. The study protocol was approved by the Ethics Committee of the Second People’s Hospital of Shenzhen (approval number: 20210812003-FS01) and registered on the Clinical Trial Register Center website: clinicaltrials.gov on January 31, 2022 (registration no.: NCT05214638).

### Participants

The study participants included patients with stroke, health professionals, and caregivers. Inpatients who met the inclusion criteria were recruited using convenient sampling facilitated by in-hospital advertising by the rehabilitation departments of the two institutions. The patients’ ADLs were assessed by health professionals and caregivers. The health professionals comprised physiotherapists, occupational therapists, and speech and language therapists employed at the two institutions. Health professionals were invited via internal email communication, and those who volunteered and met the inclusion criteria were recruited. All health professionals were skilled in the Longshi evaluation, given that the Longshi Scale was used in these two institutions. All health professionals were given brief, half‐day training sessions on how to use the WeChat version of the Longshi Scale and were informed of the study’s purpose before its commencement. The caregivers were primarily the families (or hired personnel in cases where the patients’ families were unavailable) of the recruited patients. The study procedure was explained to them, and their voluntary informed consent was obtained. The research assistants in this study were trained panelists on the research team, which facilitated a seamless study process.

The inclusion and exclusion criteria were as follows:

1. Patients with stroke:

The inclusion criteria were age of ≥ 18 years, diagnosis of cerebral infarction or intracerebral hemorrhage, stable vital signs, and willingness to participate in the study with the provision of informed consent.

The exclusion criteria were the presence of other neurological diseases, inability to cooperate in completing the assessment, and participation in any other clinical study.

2. Health professionals:

The inclusion criteria were 1–10 years of work experience, more than 2 years of experience in neurorehabilitation research or education-related work, and willingness to participate in the study with the provision of informed consent.

The exclusion criterion was the inability to complete the full assessment process.

3. Caregivers:

The inclusion criteria were being a family member or caregiver of the patient with stroke, having the ability to read and communicate, and having the willingness to participate in the study with the provision of informed consent.

The exclusion criterion was the inability to complete the full assessment process.

### Ethics approval and consent to participate

This study employed survey and interview methodologies as its primary research methods. The vulnerable demographic in this research comprised the older population. The research procedures ensured that the patients would not face any additional risks. They only need to receive ADL assessment on time as required. Patients may withdraw from the study at any stage of the study. Any alterations to the research protocol emerging during the study were expeditiously communicated to the ethics committee and implemented solely upon receiving approval. Throughout the research process, participants (including patients with stroke, health professionals, and caregivers) were incorporated into the study only after endorsing a hard copy of the informed consent form, signifying their comprehension of the principal objectives and fundamental procedures of this investigation.

This study was approved by the Ethics Committee of the Second People’s Hospital of Shenzhen (ethics approval number: 20210812003-FS01) and registered in Clinical Trials on January 31, 2022 (No.: NCT05214638). All inpatients and their proxies were invited to participate in the study after providing informed consent. All authors confirm that all the methods were performed in accordance with the relevant guidelines and regulations.

### Procedures

The study purpose and procedure were explained to all participants, and written informed consent was obtained prior to study commencement. The following demographic data and disease information of all the patients were recorded by health professionals: age, sex, and stroke type. The age and educational levels of both health professionals and caregivers were self-reported.

**Fig. 1:**
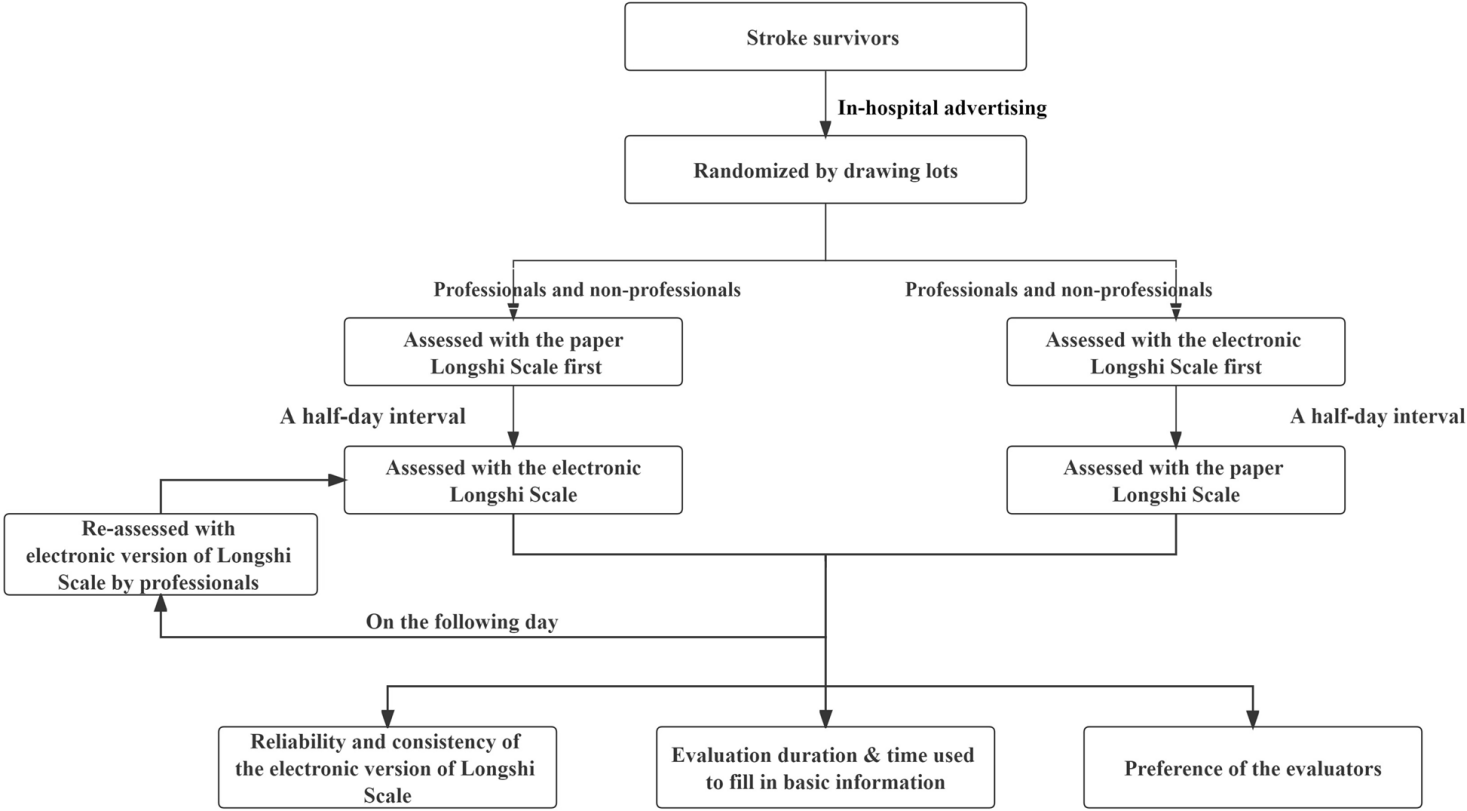
Flow diagram of the study process

**Fig. 2:**
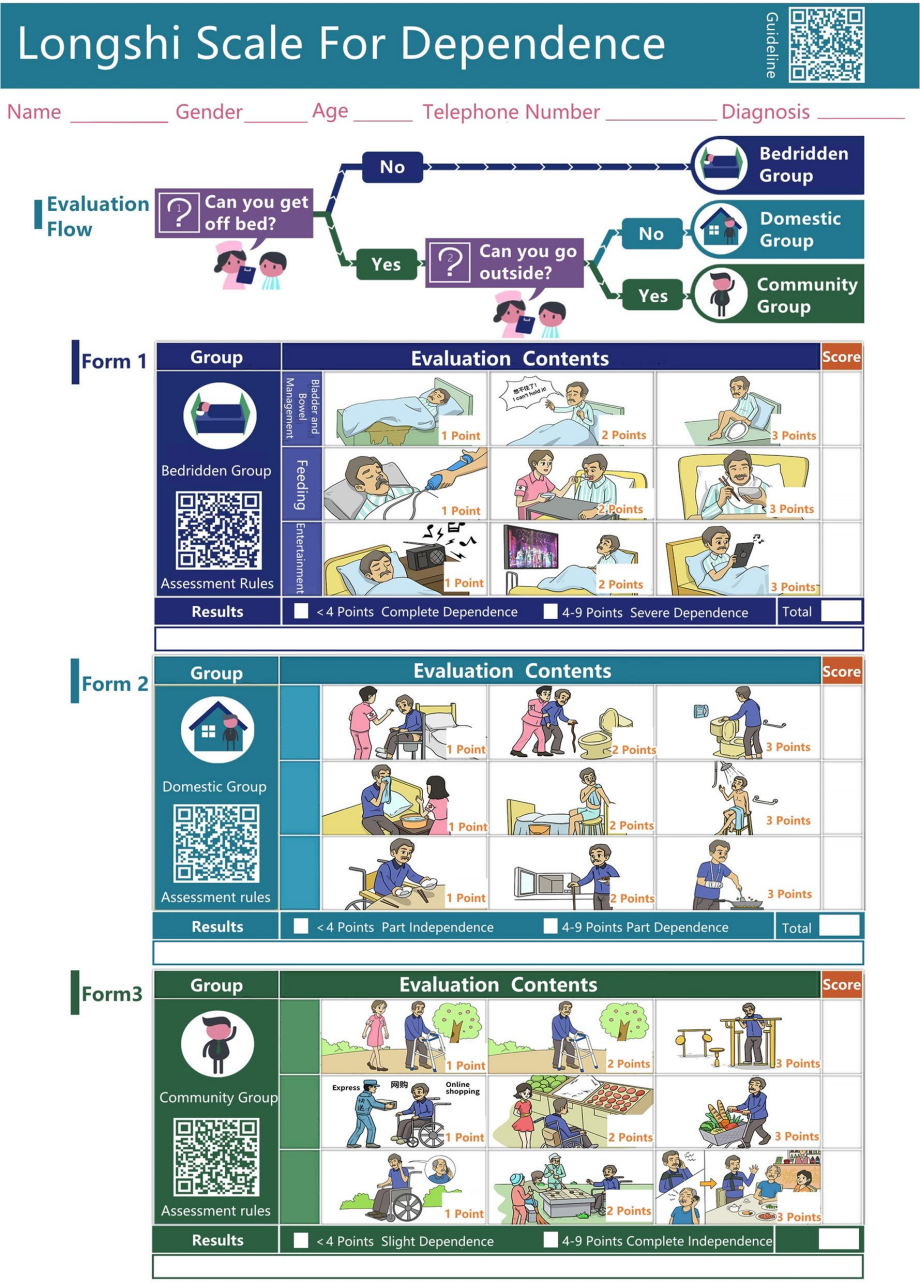
Longshi Scale for assessing ADL in individuals with disabilities

This study was conducted in the inpatient wards of two rehabilitation institutions and comprised two stages (Fig. 1). In the first stage, all patients were randomly classified into two groups (A and B) by drawing lots. The ADLs of patients in group A were independently assessed by the health professionals and caregivers during morning hours using the paper-based Longshi Scale (Fig. 2). The order of these two assessments was randomly determined by drawing lots. In the afternoon, the health professionals and caregivers conducted the electronic Longshi assessment following the same principle. A half-day interval between the paper and electronic assessments was maintained to prevent potential learning effects.

The evaluation procedure for patients in group B mirrored that of the patients in group A, with one exception: the WeChat version of the Longshi assessment was conducted in the morning, followed by the paper-based Longshi assessment in the afternoon. This order was implemented to minimize the potential impact of the evaluation sequence on the test results.

Before the ADL assessment, a research assistant and a health professional approached the patients’ bedside. The research assistant explained the entire process to the patient and their personal caregiver. To minimize interference, the research assistant stood approximately 2 m away from the evaluator and patients with stroke. The evaluator conducted the ADL assessment using either the paper-based or WeChat version of the Longshi scale. The research assistant monitored the process and recorded the duration of time used. The evaluation duration was recorded from the moment the evaluators began reading the first question to the completion of the evaluation. The research assistant also recorded the time required to fill in the basic information. Before conducting the WeChat version of the Longshi assessment, the research assistant ensured the readiness of the WeChat version of the Longshi scale for use.

The second stage of the study involved determining the test–retest reliability; 22 patients were randomly selected by drawing lots. Their ADLs were re-evaluated on the following day by a health professional who had initially assessed their ADLs using the electronic Longshi Scale.

During the paper-based Longshi assessment, the evaluation results (ADL score and degree of disability), evaluation duration, and time required to fill in the basic information were recorded; these were manually entered into the computer by research assistants. During the electronic Longshi assessment, the results were directly exported from the cloud. Once all the evaluations were complete, each evaluator was asked to disclose their preferred version.

### Paper-based version of the Longshi Scale

**Fig. 3:**
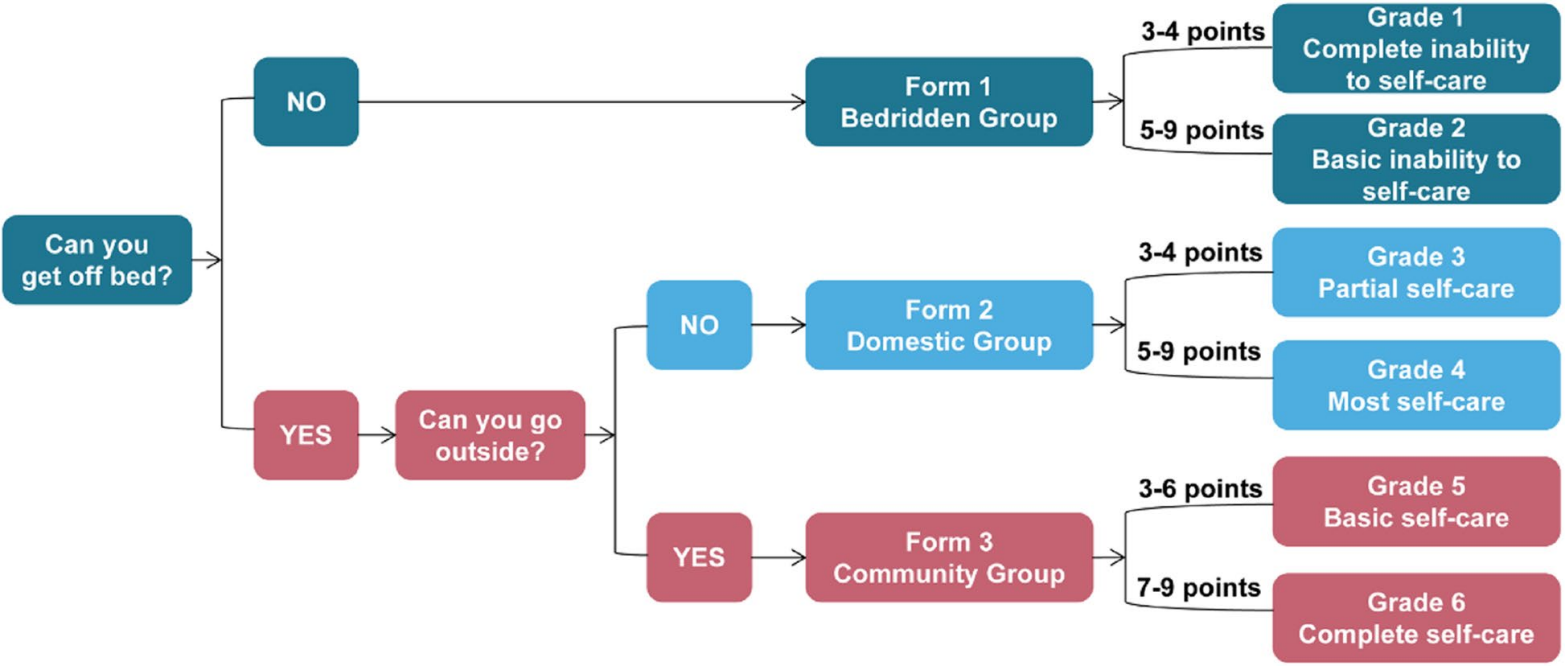
Assessment process for the Longshi Scale

The Longshi Scale was originally designed by the Rehabilitation Department of the Second People’s Hospital of Shenzhen [17]. The ADL score and degree of disability can be determined in three steps using this tool (Fig. 3). Based on the activity range, which may be restricted owing to limited physical ability, this scale first categorizes patients into three groups: i) Bedridden (patients who cannot get in or out of bed independently), ii) Domestic (patients who can get in and out of bed and can mobilize in their home environment; however, they cannot move outdoors independently with or without assisting devices), and iii) Community (patients who can mobilize outdoors independently with or without assisting devices).

Next, three ADL items were evaluated for each Longshi group. Bladder and bowel management, feeding, and leisure activities were assessed in the Bedridden group. Toileting, grooming, and housework were assessed in the Domestic group. Community mobility, shopping, and social participation were assessed in the Community group. Each item was scored from 1 to 3 (1: total dependence, 2: partial independence, and 3: total independence). The ADL score of the patients in each group was the sum of the scores of the three relevant items, ranging from 3 (minimum) to 9 (maximum).

Finally, each participant’s degree of disability was determined according to the group to which they belonged and their ADL scores in that group. Among bedridden patients, those with ADL scores of 3 and 4–9 were deemed to have the first (complete dependence) and second (severe dependence) degrees of disability, respectively. Among domestic patients, those with ADL scores of 3 and 4–9 were deemed to have the third (partial independence) and fourth (partial dependence) degrees of disability, respectively. Among community patients, those with ADL scores of 3 and 4–9 were deemed to have the fifth (partial dependence) and sixth (complete independence) degrees of disability, respectively. Thus, the first degree corresponds to total dependence, whereas the sixth degree corresponds to total independence.

### WeChat version of the Longshi Scale

**Fig. 4:**
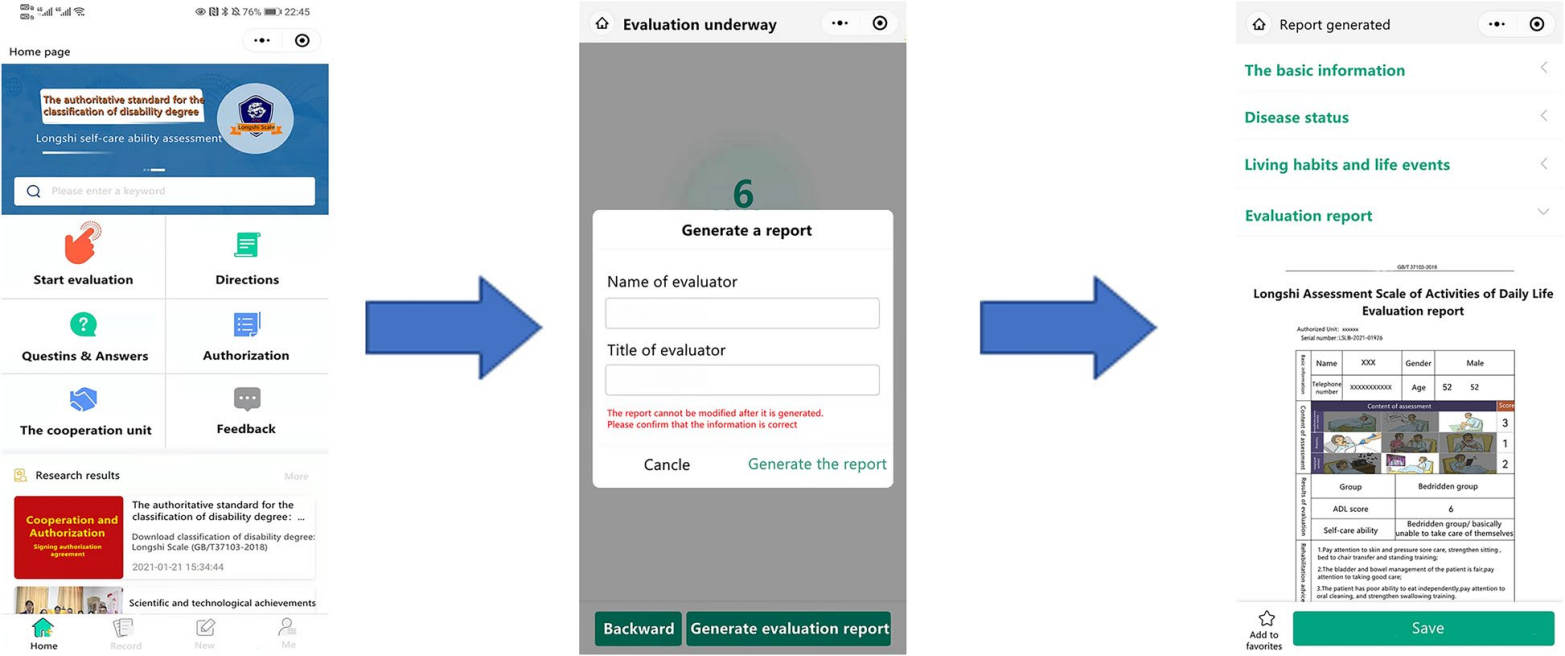
Operation process of the WeChat version of the Longshi scale

The ADL evaluation process using the WeChat version of the Longshi Scale was similar to that described for the paper-based version. The evaluation items were consistent with those of the paper-based version, with the only exception being the manner of presentation (i.e., smartphone- and paper-based). Evaluators entered “Longshi Scale: the authoritative standard for the classification of disability” in the search bar of WeChat and clicked on “start” to begin the evaluation. The assessment was conducted in a stepwise manner with voice prompts, and the system automatically directed the users to the corresponding assessment group based on their selection to avoid interference (Fig. 4). For example, when a patient was classified into the Bedridden group, they were only shown the assessment items relevant to the group. Upon completion of the evaluation, the WeChat version automatically displayed the ADL score and degree of disability, providing rehabilitation recommendations accordingly. The final diagnostic opinion and evaluation report were automatically sent to the evaluator on WeChat. Furthermore, the electronic system recorded and saved all the information and evaluation data online for documentation.

### Statistical analysis

Data were analyzed using the IBM SPSS Statistics for Windows, version 26.0 (Armonk, NY: IBM Crop); *P* < 0.05 was considered significant. The demographic and disease data, including sex, age, and stroke type, were analyzed using descriptive statistics. An independent *t*-test was used to analyze the time required for each session. The chi-square test was used to compare the proportions of the evaluators’ preferences. Linear weighted kappa (κw) values were used for the following: i) to determine the consistency between the degrees of disability evaluated by the health professionals and caregivers in both the electronic and paper-based Longshi assessments, and ii) to determine the test–retest reliability of the WeChat version of the Longshi Scale. ICC_2,1_ was used to test the inter-rater reliability of the ADL scores evaluated within each group [18]; values ≥ 0.700 were considered indicative of an acceptable level of reliability [19]. The thresholds for kappa reliability were as follows: poor (κ = 0.000–0.200), fair (κ = 0.210–0.400), moderate (κ = 0.410–0.600), good (κ = 0.610–0.800), and very good (κ = 0.810–1.000) [20].

## Results

Overall, 115 patients aged 23–88 years were enrolled in this study. The demographic data and clinical characteristics of the patients are listed in Table 1, and the ADL scores and degrees of disability are listed in Table 2.

**Table 1:** Demographic data and clinical characteristics of the patients

Characteristic	Statistics
Age, years, mean (SD)	60.500 (14.000)
Sex, n (%)	
Male	82 (71.3)
Female	33 (28.7)
Stroke type, n (%)	
Cerebral hemorrhage	47 (40.9)
Cerebral infarction	68 (59.1)

SD Standard deviation

**Table 2:** ADL scores and degrees of disability in each group assessed using the Longshi Scale

	WeChat version of the Longshi Scale	Paper-based version of the Longshi Scale
ADL score, mean (SD)		
Health professionals		
Bedridden group	4.650 (1.960)	4.710 (2.010)
Domestic group	5.300 (1.880)	5.220 (1.890)
Community group	6.500 (2.000)	6.330 (2.120)
Caregivers		
Bedridden group	4.420 (1.800)	4.490 (1.910)
Domestic group	5.700 (2.040)	5.300 (1.960)
Community group	6.210 (2.300)	6.460 (2.250)
Degree of disability, n (%)		
Health Professionals		
First degree	28 (24.3)	30 (26.1)
Second degree	30 (26.1)	31 (27.0)
Third degree	5 (4.3)	8 (7.0)
Fourth degree	25 (21.7)	23 (20.0)
Fifth degree	3 (2.6)	3 (2.6)
Sixth degree	24 (20.9)	20 (17.4)
Caregivers		
First degree	25 (21.7)	25 (21.7)
Second degree	29 (25.2)	28 (24.3)
Third degree	6 (5.2)	8 (7.0)
Fourth degree	25 (21.7)	25 (21.7)
Fifth degree	5 (4.3)	6 (5.2)
Sixth degree	25 (21.7)	23 (20.0)

*ADL* Activities of daily living, *SD* Standard deviation

### Consistency of assessment results

**Fig. 5:**
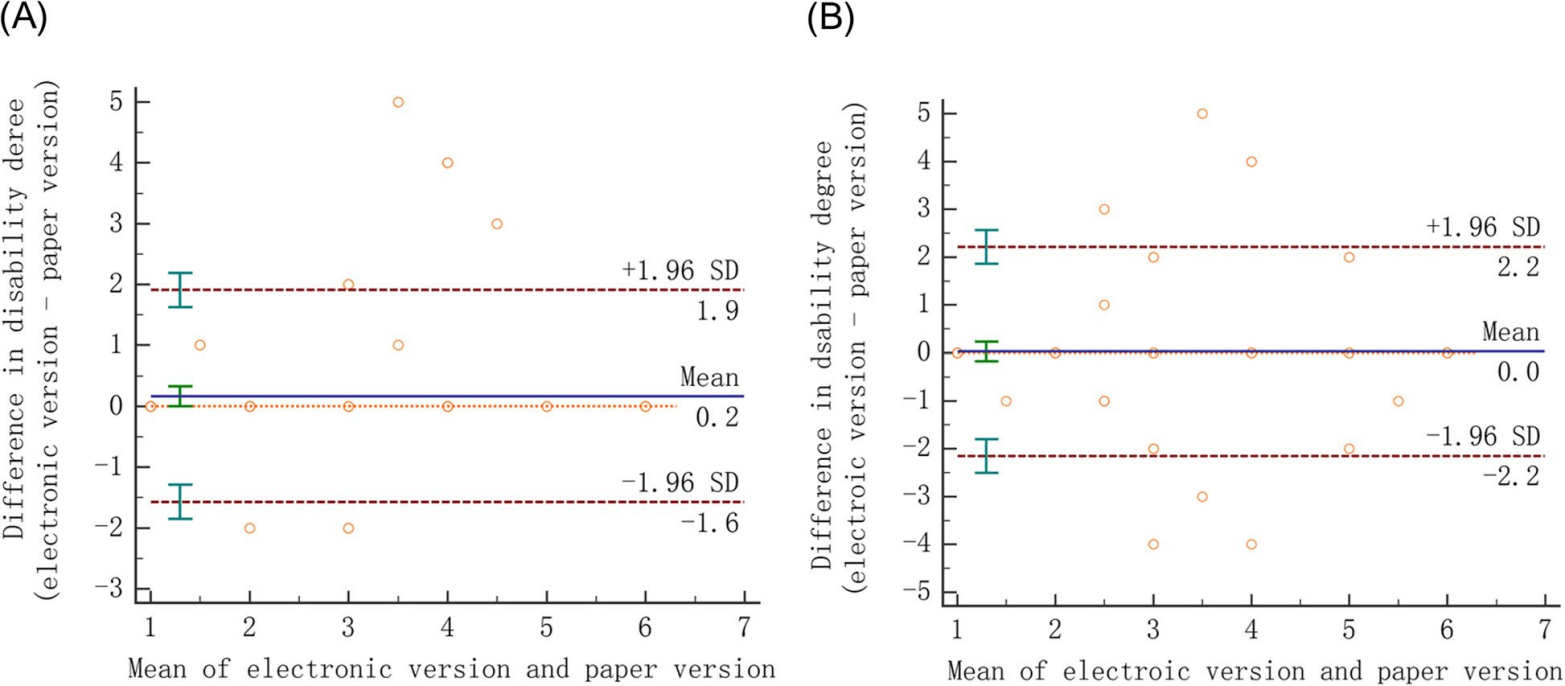
Bland–Altman analysis of agreement regarding the degree of disability evaluated using the electronic and paper versions of the Longshi Scale. **A** Degree of disability evaluated by health professionals; **B** Degree of disability evaluated by caregivers. The middle horizontal line represents the mean difference, and the upper and the lower horizontal lines represent the 95% limits of agreement (± 1.96 SD)

Table 3 presents the consistency between the electronic and paper-based versions of the Longshi Scale. The ADL scores obtained using both versions showed high consistency, regardless of whether they were obtained by health professionals (ICC_2,1_ = 0.803–0.988) or caregivers (ICC_2,1_ = 0.845–0.983). The degrees of disability evaluated using both versions were also highly consistent when assessed by both health professionals (κw = 0.870) and caregivers (κw = 0.800). The Bland–Altman analysis revealed no large discrepancies between the degrees of disability evaluated using the electronic and paper-based versions of the Longshi Scale (Fig. 5). The WeChat version also elicited good test–retest reliability (κw = 0.880).

**Table 3:** Consistency between the electronic and paper-based versions of the Longshi Scale

	Consistency of two versions by health professionals	Consistency of two versions by caregivers
ADL scores, ICC_2,1_ (95% CI)		
Bedridden group	0.988 (0.980–0.993)	0.845 (0.736–0.911)
Domestic group	0.979 (0.955–0.990)	0.983 (0.961–0.992)
Community group	0.803 (0.597–0.909)	0.919 (0.823–0.964)
Degree of disability, κw (95% CI)	0.870 (0.790–0.940)	0.80 (0.710–0.900)

*ADL* Activities of daily living, *CI* Confidence interval, *ICC*_*2,1*_ Intraclass correlation coefficient based on a two-way random effect, *κw* Linear weighted Kappa value

### Inter-rater reliability of the WeChat version

As shown in Table 4, compared with the paper-based version (ICC_2,1_ = 0.717–0.915), the WeChat version exhibited similar inter-rater reliability between the health professionals and caregivers in terms of the ADL score evaluation when compared with the paper version (ICC_2,1_ = 0.781–0.941). Both versions demonstrated good inter-rater reliability between the health professionals and caregivers (κw for the WeChat version = 0.880; κw for the paper-based version = 0.760) in terms of the degree of disability assessment.

**Table 4:** Inter-rater reliability of the Longshi Scale between the health professionals and caregivers

	Inter-rater reliability of the WeChat version	Inter-rater reliability of the paper-based version
ADL scores, ICC_2,1_ (95% CI)		
Bedridden group	0.798 (0.672–0.878)	0.849 (0.747–0.912)
Domestic group	0.941 (0.873–0.974)	0.915 (0.821–0.961)
Community group	0.781 (0.566–0.897)	0.717 (0.437–0.870)
Degree of disability, κw (95% CI)	0.880 (0.820–0.940)	0.760 (0.660–0.860)

*ADL* Activities of daily living, *CI* Confidence interval, *ICC*_*2,1*_ Intraclass correlation coefficient based on a two-way random effect, *κw* Linear weighted Kappa value

### Evaluation duration of the two versions

Health professionals required 58 ± 42.950 and 69 ± 48.760 s to complete the electronic and paper-based versions of the Longshi Scale, respectively, showing no significant differences (*P* = 0.082). Caregivers required 107 ± 58.480 and 121 ± 98.810 s to complete the electronic and paper-based versions of the Longshi scale, respectively, showing no significant differences (*P* = 0.199). However, the time spent to fill in the basic information was significantly shorter with the WeChat version than with the paper version (*P* < 0.001, 95% confidence interval [CI]: –43.463 to –15.488). Furthermore, the evaluation duration was significantly shorter for health professionals than for caregivers, regardless of whether they used the WeChat version (*P* < 0.001, 95% CI: –62.042 to –35.366) or the paper-based version (*P* < 0.001, 95% CI: 31.636 to 72.208) (Table 5).

**Table 5:** Time required to evaluate the patients using the electronic and paper-based versions of the Longshi Scale

	Evaluation duration with the paper-based version (s)	Evaluation duration with the WeChat version (s)	*P*_1_ value
Health Professionals (mean ± SD)	69 ± 48.760	58 ± 42.950	0.082
Caregivers (mean ± SD)	121 ± 98.810	107 ± 58.480	0.199
Time required to fill in basic information (mean ± SD)	105 ± 36.830	76 ± 60.920	< 0.05
P_2_ value	< 0.05	< 0.05	-

*SD* Standard deviation, *P*_1_ value: comparison between the paper-based and WeChat versions, *P*_2_ value: comparison between the health professionals and caregivers

### Preference of the evaluators

As shown in Table 6, 53.6% (15/28) of the health professionals preferred the WeChat version, whereas only 25% (7/28) preferred the paper-based version (*P* < 0.05); the remaining 21.4% (6/28) reported no preference. Conversely, 39.4% (41/104) and 33.7% (35/104) of the caregivers preferred the paper-based and WeChat versions, respectively; the remaining 26.9% (28/104) reported no preference (*P* = 0.106). The number, age, and educational level of the health professionals and caregivers are summarized in Table 7.

**Table 6:** Preference of the evaluators

	WeChat version n (%)	Paper-based version n (%)	No preference n (%)	*P*_1_ value	*P*_2_ value
Health Professionals (*n* = 28)	15 (53.6)	7 (25)	6 (21.4)	< 0.05	< 0.05
Caregivers (*n* = 104)	35 (33.7)	41 (39.4)	2 (26.9)	0.160	0.388

*P*_1_ value: comparison among the three options; *P*_2_ value: comparison between the electronic and paper-based versions

**Table 7:** Age and educational level of health professionals and caregivers

	Health professionals (*n* = 28)	Caregivers (*n* = 115)
Age, n (%)		
20–30	9 (32.1)	3 (2.6)
30–40	17 (60.7)	12 (10.4)
40–50	2 (7.1)	39 (33.9)
50–60	0 (0)	51 (44.3)
60–70	0 (0)	6 (5.2)
70–80	0 (0)	3 (2.6)
80–90	0 (0)	1 (0.9)
Educational level, n (%)		
Elementary level or blew	0 (0)	33 (28.7)
Secondary & High school	0 (0)	63 (54.8)
Bachelor degree	26 (92.9)	19 (16.5)
Master degree	2 (7.1)	0 (0)

## Discussion

In the current study, we examined the implementation of the WeChat version of the Longshi Scale by comparing it with the paper-based version. We found that the WeChat version has good reliability and was highly consistent with the paper-based version, regardless of whether it is used by health professionals or caregivers. Furthermore, WeChat allowed caregivers to accurately assess patients’ ADL without the need for professional medical training.

Electronic health management is well aligned with traditional paper-based approaches in patient management. A meta-analysis revealed that the electronic and paper-based versions of patient-reported outcome measures were equivalent and highly consistent with each other (average ICC = 0.900) [21]. As expected, the WeChat version of the Longshi scale had good inter-rater reliability and was highly consistent with the paper-based version during the evaluation of the ADL scores and degree of disability, regardless of whether the user was a health professional or caregiver. Sun et al. compared the evaluation results from the WeChat and paper-based versions of the Pelvic Floor Impact Questionnaire-Short Form 7. Similar to our findings, the authors found that the scores from all three subscales of this questionnaire assessed using the WeChat version were highly consistent with those of the paper-based version (ICC = 0.915–0.980) [22]. The electronic and paper-based versions of the health management tools examined in both the aforementioned meta-analysis [21] and our study were highly consistent, even though the electronic platforms used differed. This implies that the WeChat versions of evaluation systems can achieve reliable results that are consistent with those of other WeChat versions.

In addition, we found that the consistency of evaluation results between caregivers and health professionals revealed similar inter-rater reliability when using the WeChat version of the Longshi Scale compared with the paper-based Longshi Scale. Interestingly, the accuracy of the results reported in other studies on WeChat versions of evaluation systems varies. Koo et al. found that written records of patient handovers were prone to errors or incompleteness and that the accuracy depended on the person entering the information. Handover printouts automatically generated from electronic health records (EHR) can reduce the risk of transcription errors and improve formatting consistency [23]. Conversely, Moomba et al. reported a decrease in the completeness and accuracy of vital sign changes with electronic recording [24]. A plausible explanation for the higher accuracy in our study is that the voice prompts in WeChat help caregivers in the evaluation process; the stepwise evaluation process itself guides these evaluators (who generally do not have a comprehensive understanding of the scale) to complete the evaluation without confusion. This may indicate that individuals with limited medical backgrounds can accurately assess their patients’ ADL, even without professional training.

Herein, the evaluators completed the WeChat version of the Longshi Scale in less time than the paper version, although the difference was not statistically significant. This result is in accordance with that of a study using the short form-36, in which the electronic form was completed in less time than the paper form, and the difference was not statistically significant [5]. However, the WeChat version of the Longshi Scale can simplify data collection and documentation, and the evaluation results can be exported automatically at the backend. This saves time and prevents potential errors resulting from manual entry of the results into the computer [25]. The WeChat version of the Longshi Scale is well accepted among allied healthcare workers, and this trend is consistent with that reported in other studies on electronic healthcare management tools [26, 27]. However, individuals with limited or no medical background have different opinions regarding this format. For instance, Belisario et al. reported that, in terms of the preference between electronic and paper instruments, more patients (48%) felt satisfied with the electronic version [28]. However, in our study, there was no significant difference in the preference of caregivers (33.7% for the WeChat version vs. 39.4% for the paper-based version). Educational level and age are potential reasons for this observation. Zhang et al. reported that 50% of patients preferred e-questionnaires, whereas only 15% preferred paper-based forms [29]. This was expected, given that a higher proportion of their patients had received higher education and only 30% had an educational level below high school; however, up to 81% of our caregivers had an educational level of high school or below. We also examined age as a factor. More than 70% of the caregivers in our study were aged 40–60 years, which may explain the lower acceptance of electronic forms, as younger patients with a higher educational level are more likely to use digital methods in healthcare management [30].

Our study demonstrated that caregivers with limited or no medical background could accurately assess patients’ ADL using the WeChat Longshi Scale. Thus, it may be possible to reduce the number of rehabilitation professionals needed in economically underdeveloped areas and allow individuals in rural areas to receive appropriate rehabilitation services. Recently, WeChat has established a large user base in China, which plays an important role in health promotion and chronic disease management [31]. WeChat, the most popular messaging and social media platform in China, has become a vital part of Chinese citizens’ daily lives [32, 33]. Moreover, China has continued to strengthen the construction of its Internet infrastructure in rural areas. It was anticipated that by 2022, all existing administrative villages in China would have broadband coverage, and the Internet penetration rate in rural areas would reach 58.8% [34]. The prevalence of Internet use has resulted in the widespread use of WeChat across China, both in urban and rural areas. This allows less-trained medical workers or even volunteers in remote areas to accurately evaluate ADL using the WeChat Longshi Scale to provide appropriate care to clients with disabilities.

WeChat was selected as the mobile platform for developing the Longshi Scale. Compared with electronic medical records (EMR) and EHR, which are common electronic health management tools used internationally, WeChat has advantages in China owing to its popularity and has received initial positive feedback. EMR and EHR are disruptive electronic processes that can improve the quality and reliability of healthcare service delivery [35, 36]. However, programming and data extraction are time-consuming, and specialized expertise and iterations are required to rectify issues encountered during implementation [4]. Mobile health management has become a trend that has redefined the healthcare landscape [37].

With easy-to-use and convenient data storage and extraction processes, mobile health management can complement EMR and EHR. However, considering the limited use of WeChat globally, it is difficult to promote and implement the WeChat Longshi Scale on a global scale. Therefore, we plan to develop a mobile app version of the Longshi Scale to encourage its use in additional countries.

Nevertheless, the present study has some limitations. Our research was conducted in rehabilitation departments, and the caregivers were generally older and had poor educational backgrounds. This may have affected the acceptance of electronic devices and relevant products. Future studies should include a wider population sample. Our research was conducted in urban areas; therefore, additional studies need to be conducted in rural areas to examine the implementation of the WeChat Longshi Scale.

## Conclusions

Our findings suggest that the WeChat version has good reliability, is highly consistent with the paper-based version, and allows caregivers to accurately assess patients’ ADL without medical training.

## Data Availability

The datasets used and/or analyzed in the current study are available from the corresponding author upon reasonable request.

## Abbreviations

Κw: Linear weighted kappa
ADL: Activities of daily living
BMI: Body mass index
ICC_2,1_: Intraclass correlation coefficient based on two-way random effect
